# The genus *Erechthias* Meyrick of Ascension Island, including discovery of a new brachypterous species (Lepidoptera, Tineidae)

**DOI:** 10.3897/zookeys.341.6146

**Published:** 2013-10-07

**Authors:** Donald R. Davis, Howard Mendel

**Affiliations:** 1 Department of Entomology, National Museum of Natural History, Smithsonian Institution, P.O.Box 37012, MRC 105, Washington, D.C. 20013-7012, USA; 2 Department of Life Sciences (Entomology), Natural History Museum, Cromwell Road, London SW7 5BD, UK

**Keywords:** Brachyptery, distribution, genital morphology, larval case

## Abstract

One previously named and two new species of the tineid genus *Erechthias* Meyrick are described and illustrated from the small, remote, mid-Atlantic Ascension Island. With these additions the Lepidoptera fauna of Ascension now totals 38 known species. Little is known regarding the biology of the two new species of *Erechthias*, and none of the species has been reared from larvae from Ascension. *Erechthias minuscula* (Walsingham) is a widespread, largely pantropical species first described from the West Indies. Larvae of *Erechthias minuscula* are known to be scavengers on a wide variety of dead plant material. *Erechthias ascensionae*,new species, is one of two species of *Erechthias* now known to be endemic to the island. The other endemic species, *Erechthias grayi*, new species, is further remarkable in having wing reduction occurring in both sexes. It is one of the few species of Lepidoptera known where this extreme of brachyptery involving both sexes has evolved. The larvae of *Erechthias grayi* are believed to be lichenivorous, and larval cases suspected to represent this species are illustrated.

## Introduction

**Figures 1–3.**
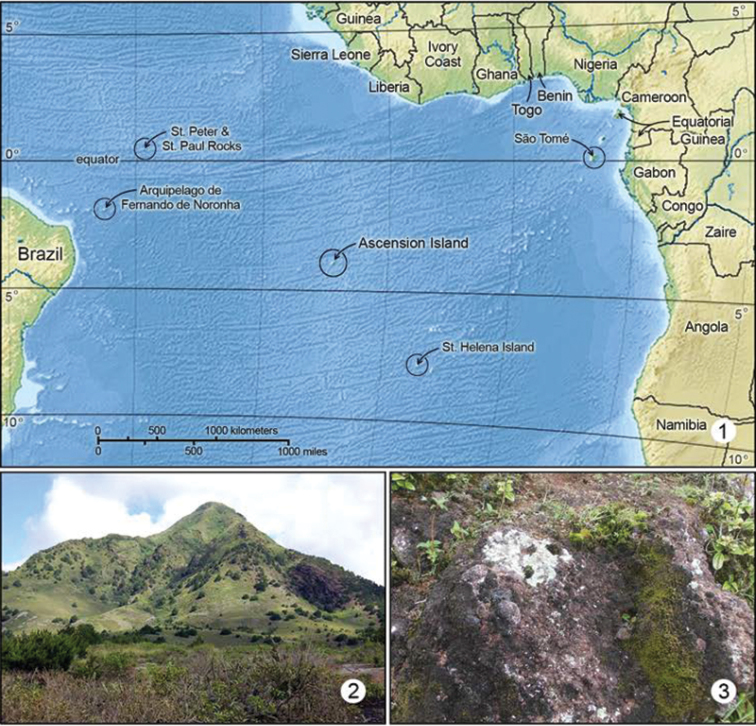
**1** Map of mid Atlantic Ocean. Ascension Island habitats: **2** Green Mountain **3** Typical habitat of *Erechthias grayi*; Lichen covered rocks on Green Mountain.

Ascension is a small, remote, tropical, volcanic island located in the middle of the Atlantic Ocean (Fig. 1), on the Mid-Atlantic Ridge. At 7°57'S, 14°22'W, it is some 1500 km from Africa and 2000 km from South America. The nearest land is St. Helena, another small, volcanic island, 1300 km to the south-east. Ascension Island comprises about 98 sq km of volcanic deposit, with a maximum elevation above sea level of 860 m.

According to Chase and Manning (1972) Ascension Island arose during either the Pliocene or Pleistocene. The most recent phase of volcanism may have occurred less than 1000 years ago (Packard 1983). The oldest rocks above sea level date to only about one million years ago, and Ashmole and Ashmole (2000) estimate this to be the age of the island. This appears to be the comparatively short time frame within which evolutionary processes have been able to work on the island’s colonists. A detailed treatment of the geology of Ascension is provided by Weaver (1999).

The island of Ascension was discovered on Ascension Day in 1501 by the Portuguese explorer and sea captain João da Nova, but there was no settled population for another three centuries, probably because of the lack of permanent fresh water. Settlement began in 1815 when the British established a garrison to prevent the French navy using the island in any attempt to free Napoleon Bonaparte from St. Helena, where he had been exiled after the Battle of Waterloo. Napoleon never escaped and died on St. Helena in 1821.

The climate is tropical but the oceanic position of the island and the influence of the south-east trade winds are huge modifying influences. Although Green Mountain (Fig. 2) rises to a mere 860 m, it is cool, damp, windy, and frequently shrouded in mist and capped with cloud. It is indeed green compared with most of the island, where the vegetation is sparse and near-desert conditions prevail. However, the vegetation we see today across the island has been highly modified by deliberate and accidental introductions, and the impact of introduced animals such as goats, sheep and donkeys. Surviving areas of near natural vegetation are mostly difficult to access.

For this brief introduction, we have principally relied on information from Duffey (1964), Robinson and Kirke (1990), Disney (1991) and Ashmole and Ashmole (2000).

Robinson and Kirke (1990), who reviewed the Lepidoptera fauna of Ascension Island, concluded that it was ‘remarkable only for its impoverishment, consisting of a small range of pantropical and pest species’. They recorded a total of 37 species, and with the possible exception of two species of Tineidae, a *Eudarcia* sp. and an *Erechthias* sp., they found no endemism. Most of the Lepidoptera they studied appeared to have originated from the Afrotropical and Mediterranean regions, either by introduction or immigration.

*Erechthias* is the largest, most diverse genus within the tineid subfamily Erechthiinae. Approximately 120 described species of *Erechthias* have been reported, with almost as many still undescribed (Robinson and Nielsen 1993). The great majority of the species are Old World in distribution. In the present study we describe and illustrate the one previously named (*Erechthias minuscula*) and one unnamed (*Erechthias ascensionae*) species of *Erechthias* that Robinson (2009) reported from Ascension Island. *Erechthias minuscula* (Walsingham) is a widespread, largely pantropical species first described from the West Indies. *Erechthias ascensionae*,new species, is one of two species of *Erechthias* known to be endemic to the island. As Robinson (2009) had suspected, this species appears morphologically most similar to *Erechthias dracaenura* (Meyrick), described from one of the nearest islands to Ascension, São Tomé, which is located off the western coast of central Africa in the Gulf of Guinea. We also report the discovery of a brachypterous species, *Erechthias grayi*, new species, which is further remarkable in having wing reduction occurring in both sexes. This species was mentioned by Robinson (2009) in his brief review of the Ascension Island Tineidae, but he was uncertain of its generic and subfamily affinities.

## Material

Specimens examined in this study are deposited in the following institutions.

BMNH Natural History Museum, formerly British Museum (Natural History), London, United Kingdom.

USNM Collections of the former United States National Museum, now deposited in the National Museum of Natural History, Smithsonian Institution, Washington, D.C., USA.

## Methods

### Specimen preparation

Genitalic dissections were cleared by heating in 10% KOH for ~ 30 minutes, and subsequently cleaned and stained with either 2% chlorazol black E or mercurochrome solutions. The genitalia were then mounted on slides using Canada balsam or euparal mounting media. Genitalic terminology follows Klots (1970).

## Systematic account

### Erechthias

Meyrick

http://species-id.net/wiki/Erechthias

Erechthias Meyrick, 1880: 252, 261. Type species: *Erechthias charadrota*Meyrick 1880: 268 by subsequent designation by Meyrick 1915: 233. [New Zealand].
Ereunetis Meyrick, 1880: 252, 258. Type species: *Ereunetis iuloptera*Meyrick 1880: 258, 260, by subsequent designation by Walsingham 1914: 347. [Australia].
Decadarchis Meyrick, 1886: 290. Type species: *Decadarchis melanastra*Meyrick 1886: 291, by monotypy. [Fiji].
Hactacma Meyrick, 1915: 233. Type species: *Erechthias chasmatias*Meyrick 1880: 263, 264, by original designation. [New Zealand].
Nesoxena Meyrick, 1929: 506. Type species: *Nesoxena strangulata*Meyrick 1929: 507. [Tuamotu Archipelago].
Amphisyncentris Meyrick, 1933: 412. Type species: *Amphisyncentris glyphidaula*Meyrick 1933: 412, by monotypy. [Fiji].
Gonglyodes Turner, 1933: 180. Type species: *Gonglyodes centroscia*Turner 1933: 180, by monotypy. [Australia].
Caryolestis Meyrick, 1934: 109. Type species: *Caryolestis praedatrix*Meyrick 1934: 110, by monotypy. [Tahiti].
Triadogona Meyrick, 1937: 153. Type species: *Triadogona amphileucota*Meyrick 1937: 153, by monotypy. [Fiji].
Anemerarcha Meyrick, 1937: 154. Type species: *Anemerarcha entomaula*Meyrick 1937: 154, by monotypy. [Fiji].
Empaesta Bradley, 1956: 163. Type species: *Tinea capnitis*Turner 1918: 288, by original designation. [Norfolk Island].
Tinexotaxa Gozmány, 1968: 306. Type species: *Tinexotaxa travestita*Gozmány 1968: 306, by original designation. [Sierra Leone].
Acrocenotes Diakonoff, [1968]: 259, 262. Type species: *Acrocenotes niphochrysa*Diakonoff [1968]: 257, 262, by original designation. [Philippines].
Neodecadarchis Zimmermann, 1978: 264, 341. Type species: *Ereunetis flavistriata*Walsingham 1907: 716, by original designation. [Hawaii].
Lepidobregma Zimmermann, 1978: 264, 351. Type species: *Ereunetis minuscula*Walsingham 1897: 155, by original designation. [West Indies].
Pantheus Zimmermann, 1978: 264, 353. Type species: *Ereunetis pencillata*Swezey 1909: 13, by original designation. [Hawaii].

#### Adult.

*Head* (Figs 12–15): Frons with scales moderately broad, either mostly appressed or partially raised, sometimes with weak scale tufts arising from ventro-lateral margin; pilifers present, with numerous, short bristles; vertex with prominent occipital and lateral tufts and scales more slender; Eye of medium size; frons broad; interocular index (Davis 1975) 0.7–1.2. Antenna extending ~ 0.7–1.0× length of forewing; scape with prominent pectin of ~10–14 bristles (Fig. 13); intercalary sclerite well sclerotized; flagellomeres with a single annulus of appressed, narrow scales; antennal cilia short in both sexes. Maxillary palpus as long as or usually slightly longer than labial palpus, 5-segmented, with length ratio of segments from base: 1.0: 0.5: 1.7–2.75: 5.5–8.7: 2-3.6. Haustellum moderately developed, ~ 0.6–1.0× length of labial palpus. Labial palpus well developed, length ratios from base: 1.0: 1.3–2.5: 1.0–1.4; segment 2 sometimes broad at base, with a prominent ventral brush of elongate, slender scales; a series of 5-16 long whitish to black bristles arising mostly laterally; 1-3 long, lateral bristles also usually present on basal segment.

*Thorax*: Wings (Figs 16–18) relatively narrow; forewing L/W index: 0.23–0.27; hindwing L/W index: 0.24–0.32. Forewing with Sc and R present in all species, Rs usually with 3–4 branches, reduced to one branch in *Erechthias grayi*; Rs4 and M1 rarely stalked; accessory cell usually absent, sometimes with a trace of chorda; M with 2 branches (M1 and M2+3), with M fused with Cu in *Erechthias grayi*; base of M usually absent or vestigial in cell; Cu with 2 branches (fused with M in *Erechthias grayi*); CuP usually weak; A3 vestigial; retinaculum in male on underside of subcoasta; triangular, with a rolled apex. Hindwing with Sc and R fused; Rs usually present but incomplete, unbranched; M usually 3-branched, sometimes with M1and 2 fused; Cu 2-branced; A3 usually present; frenulum with single large spine in male, 1-3 spines in female. Legs unmodified; foretibia with epiphysis arising near distal third of tibia; midtibia with a single pair of spurs of unequal length arising near apex; hindtibia with 2 pairs of spurs of unequal lengths arising near basal third of tibia and near apex.

*Abdomen*: Apodemes slender, slightly convergent, or short, basally broad and nearly triangular. Segment A8 with male coremata present or absent; female corethrogyne absent.

*Male genitalia*: Segment A10 mostly membranous, often setose and melanized laterally; apex of uncus variably bilobed. Tegumen a narrow dorsal ring, poorly differentiated from vinculum; vinculum with a well developed, usually broadly rounded, triangular saccus. Valva usually simple, rarely with lobes or processes, usually broad, and often with a dense concentration of thick, costal spines; costal apodemes usually well developed. Gnathos absent. Juxta highly modified, forming sclerotized pouch of variable depth between bases of valvae and articulating with costal apodemes of valvae. Aedeagus typically in the form of a simple cylinder, with a slightly swollen base in some species; vesica sometimes lined with minute spicules, or with 1–2 much larger, spine-like cornuti.

*Female genitalia*: Ovipositor short to moderately long, 0.05–0.50× length of abdomen; posterior ventral apophyses not developed; posterior (dorsal) apophyses 1.5–2.7× length of anterior apophyses. Eighth tergite often narrowly rectangular, with a few terminal setae; eighth sternum connected to anterior apophyses by ventral rami. Bursa copulatrix 0.5–1.9× length of abdomen. Antrum relatively slender, often narrowly funnel-shaped or sometimes in the form of an incomplete ring; junction with ductus seminalis immediately anterior to antrum. Ductus bursae slender, with or without coarse microtrichia lining interior. Corpus bursae membranous, ovate to pyriform, often with a single small signum with the more slender, rodlike end projecting free from exterior wall of corpus bursae; signum sometimes can be stellate, blade-shaped, in the shape of a small ridged plate, or absent.

#### Key to species of *Erechthias* occurring on Ascension Island

**Table:** 

1	Adult brachypterous; forewing less than 2 mm long; hindwing nearly absent, less than 0.2 mm long (Fig. 18)	*Erechthias grayi*
–	Adult fully winged; forewing more than 3 mm long; hindwing not reduced	2
2	Apex of forewing not turned sharply upwards; color pale whitish cream heavily irrorated with dark brown scales (Fig. 5)	*Erechthias ascensionae*
–	Apex of forewing sharply upturned; color pale whitish cream, variably irrorated or streaked with medium to dark brown scales (Fig. 4)	*Erechthias minuscula*

### Erechthias minuscula

(Walsingham)

http://species-id.net/wiki/Erechthias_minuscula

Figs 4 , 12–14 , 16 , 19–23

Ereunetis minuscula Walsingham, 1897: 155; 1907: 716.- Swezey 1909: 12.- Busck 1911: 80.- Swezey 1912: 155.- Walsingham 1914 [1909–1915]: 347.- Wolcott 1923: 205.- Swezey 1929: 281.- Forbes 1930: 147.- Wolcott 1936: 501.- Swezey 1940: 458.- Wolcott 1948: 739.- Beardsley 1961: 354.
Decadarchis minuscula (Walsingham).- Meyrick 1929: 505.- Harris 1937: 486.- Ghesquière 1940: 86.- Vesey-Fitzgerald 1941: 158.- Lepesme 1947: 318.- Viette 1949: 316.- Swezey 1942: 215.- Davis 1953: 85.- Swezey 1952: 378.- Diakonoff 1968: 265, 308.- Clarke 1971: 211; 1986: 361.- Zimmermann 1978: 352.
Lepidobregma minuscula (Walsingham).- Zimmermann 1978: 352.- Davis 1983: 5.- Robinson and Nielsen 1993: 289.
Erechthias minuscula (Walsingham).- Davis 1984: 21.- Robinson and Kirke 1990: 133.- Robinson 2009: 20–25, 51, fig. 36.- Robinson and Nielsen 1993: 295, 310.- Heppner 2003: 236.

#### Adult

**Figures 4–11.**
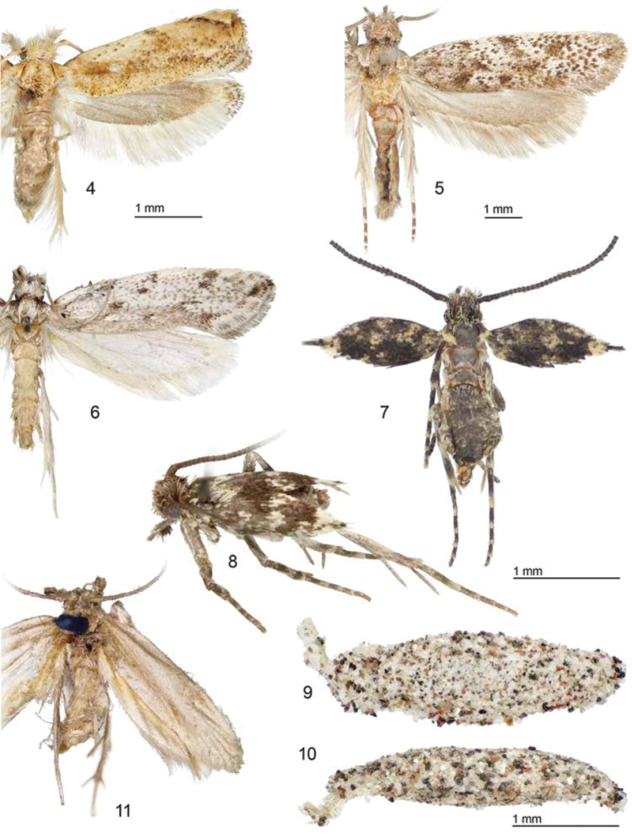
Adults and larval cases **4** ♂, *Erechthias minuscula*, (forewing length 4 mm), Florida, USA **5** ♂, *Erechthias ascensionae*, (forewing length 6.8 mm), Ascension Island **6** ♂, *Erechthias dracaenura* (forewing length 8 mm), São Tomé **7, 8** ♂, *Erechthias grayi* (forewing length 1.7 mm), Ascension Island **9** (dorsal) **10** (lateral) Larval cases *Erechthias* sp. *grayi* ? (length 4.2 mm), Ascension Island **11** ♂, *Erechthias darwini* (forewing length 6 mm), St. Paul’s Rocks.

(Fig. 4). *Head*: Scales of frons shiny whitish cream, moderately broad with 3-4-dentate apices, flatly appressed to frons with apices directed dorsad; a pair of scale tufts consisting of very slender, elongate scales arising from lower corners of frons. Vertex with a prominent pair of lateral, occipital tufts composed of very elongate, piliform, pale cream scales with minutely bidentate scales. Labial palpus with apical segment mostly smoothly scaled; scales of segment 2 flat, strongly appressed dorsally, shiny white to whitish cream; venter of segment 2 with a dense brush of long, slender, erect, whitish (rarely suffused with dark brown) scales with minutely bidentate apices, and a lateral series of ~ 13–16 long, whitish bristles sometimes with dark apices. Antenna ~ 2/3 as long as forewing; scales smoothly appressed, uniformly pale cream except for scales with dark brown apices along anterior edge and sometimes venter of flagellum; scape mostly pale cream irrorated with dark brown scales along anterior edge; pectin well developed, consisting of a longitudinal row of 12–14 long dark setae; scales of scape and pedicel moderately broad; flagellum with a single row of more slender scales completely encircling each segment; short cilia usually not evident but often visible between scales.

**Figures 12–15.**
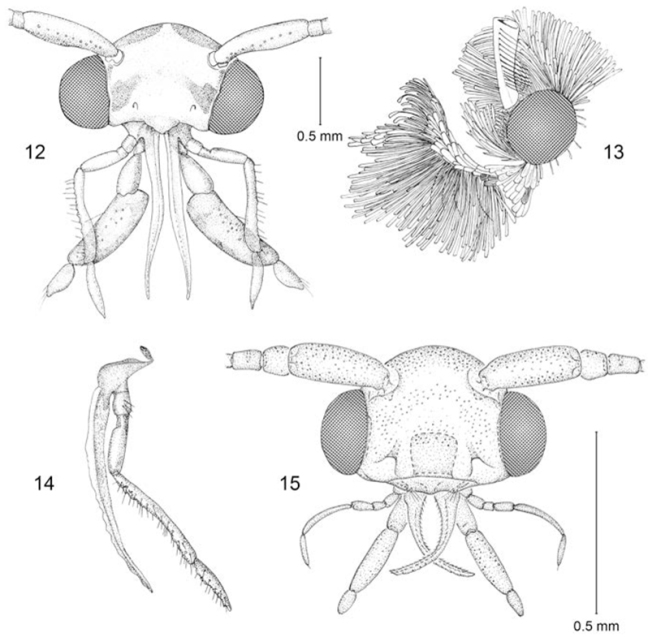
Adult head structure. **12–14** *Erechthias minuscula* **12** (anterior view) **13** (lateral view) **14** (maxilla) **15** *Erechthias grayi* (anterior view).

**Figures 16–18.**
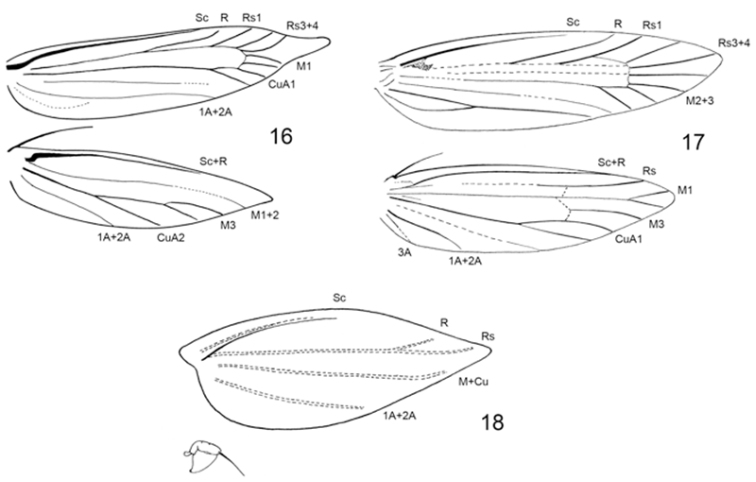
Wing venation **16** *Erechthias minuscula* **17** *Erechthias ascensionae* **18** *Erechthias grayi*.

*Thorax*: Forewing length 3.5–4.0 mm (wing apex not extended) – 5.0 mm (wing apex fully extended). Dorsum and tegula pale cream, usually with suffusion of light brown medially; apices of light brown scales often with dark brown apices giving a mottle appearance to dorsum; thorax whitish cream ventrally. Forewing predominantly pale whitish cream, variably irrorated or streaked with medium to dark brown scales; a medium to dark brown, variously interrupted streak usually extending from wing base almost to tornus (completely lacking in some specimens); costal margin often with small concentrations of dark brown scales present near base of forewing, near distal 2/3, distal 4/5 and at apex; those markings near apex sometimes forming short striae; a pale whitish cream patch of scales along margin between spot at distal 4/5 and apex; apex of forewing bent abruptly dorsad; fringe white to pale cream, variably banded at apex with 1–2 slender bands of dark brown scales; most of ventral surfaces of fore and hindwings (except for whitish cream fringes) medium to dark brown. Hindwing uniformly pale grayish brown; fringe mostly concolorous except for 2-3 narrow bands of dark brown scales around apex. Frenulum a single long spine in male and female. Fore and midlegs mostly pale to dark brown dorsally and pale cream ventrally with pale to dark brown banding on tibia and tarsomeres; hind leg generally paler, almost entirely pale cream in color.

*Abdomen*: Mostly pale golden brown dorsally, whitish cream ventrally. Eighth segment without coremata.

**Figures 19–23.**
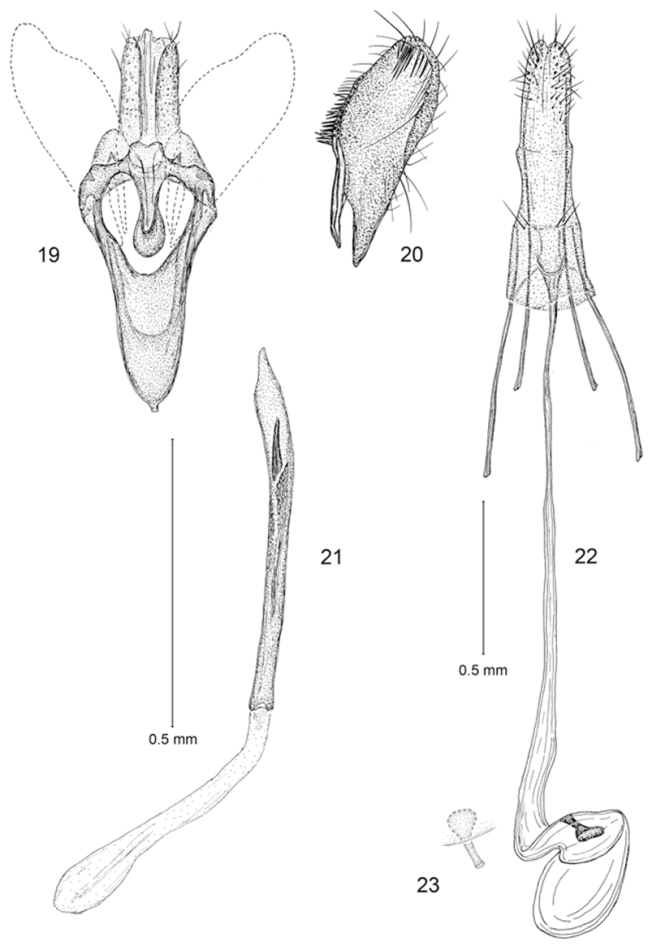
*Erechthias minuscula*, genitalia **19** Male, ventral view **20** Valva, mesal view **21** Aedeagus, ventral view **22** Female, ventral view **23** Detail of signum in Fig. **22**.

*Male genitalia* (Figs 19–21): Segment 10 mostly membranous, moderately sclerotized to form 2 elongate lateral lobes, with caudal margin superficially bifid. Tegumen a relatively narrow dorsal ring. Vinculum slender, narrowly V-shaped, elongate, ~ 0.8× the length of valva. Valva simple; cucullus broad with broadly rounded apex; costal margin densely setose, with setae concentrated near base of costa and less so at apex of valva. Juxta well developed as an elongate U-shaped pouch. Aedeagus a slender, simple cylinder, ~ 1.6× length of valva; vesica lined with numerous, minute spicules and with a single large, apical cornutus, sometimes closely accompanied with a shorter cornutus ~ half the length of the larger one.

*Female genitalia* (Fig. 22, 23): Eighth sternite weakly sclerotized; ostium opening near anterior margin, with a pair of moderately short setae on either side of ostium. Antrum reduced, triangular; length ~ equal to maximum width. Ductus bursae very slender and elongate; length ~ 1.7× that of posterior apophyses, ductus gradually enlarging to relatively small, ovate corpus bursae; walls of corpus bursae membranous except for a very small, elongate, triangular signum; distal, more slender half of signum projecting beyond wall of corpus bursae.

#### Lectotype.

♂ (present designation), WEST INDIES: Type H.T.; St. Thomas, Danish West Indies, 17 March 1894, Hedemann 7084; BM genitalia slide No. 4177; Walsingham Collection 1910-427; *Ereunetis minuscula* Wlsm., P.Z.S. p. 155 (1897), Type ♂; (BMNH).

#### Material examined.

ASCENSION ISLAND: Green Mountain: 4 ♂, 17–26 June 1988, C.M.StG Kirke, BM 1988-311, ♂ slide 29708, (BMNH).

#### Distribution.

(Fig. 1). *Erechthias minuscula* is probably pantropical in distribution, and occurs widely in South America, the West Indies into southern Florida, USA. Robinson and Nielsen (1993) also report it from Australia.

#### Biology.

Larvae of *Erechthias minuscula* are scavengers on a wide variety of dead plant material and have been reported feeding on or within dead tree trunks, stems, seed pods, fruits, flowers, and leaves (Heppner 2003: 236).

### Erechthias ascensionae sp. n.

http://zoobank.org/2FEB95B4-F5DA-4C6D-B10A-A602725E862F

http://species-id.net/wiki/Erechthias_ascensionae

**Figures 24–27.**
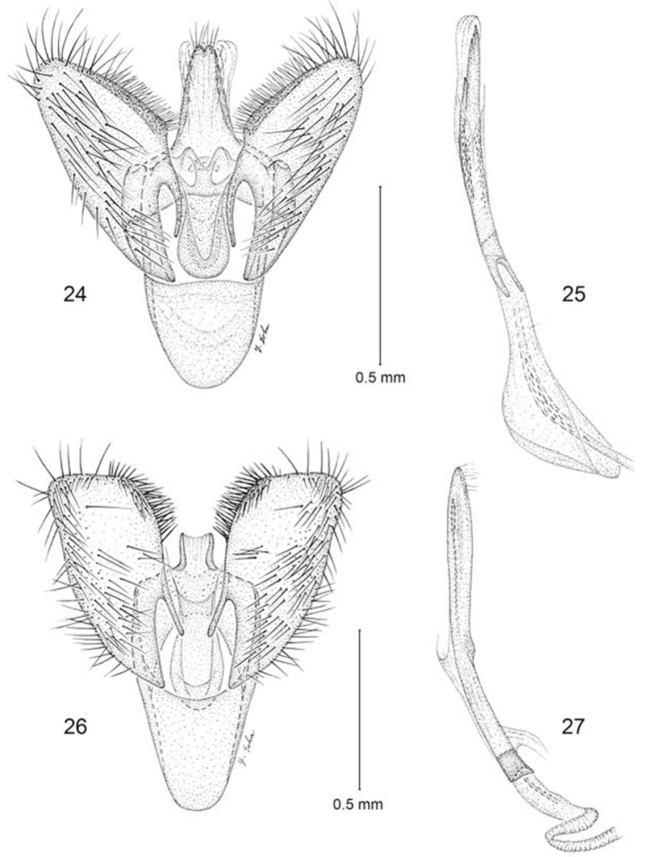
Male genitalia **24–25** *Erechthias ascensionae*, ventral view **25** Aedeagus, lateral view **26–27** *Erechthias dracaenura*, ventral view **27** Aedeagus, lateral view.

Figs 5 , 17 , 24 , 25

Ereunetis species.- Robinson and Kirke 1990: 133.- Robinson 2009: 51.

#### Diagnosis.

Female unknown.

**Adult** (Fig. 5). *Head*: Scales very slender with bidentate apices; scales of frons partially raised and directed forward, light brown strongly irrorated with dull white; scales of vertex erect or mostly so, especially over occipital tufts, and mostly directed forward; color similar to frons with most scales with dull white apices,. Labial palpus with scales slightly appressed dorsally, mostly dull white with light brown bases to scales; venter of labial palpus with a dense brush of long, slender, erect, brown scales usually with white apices, and a lateral series of ~ 5-7 long, dark bristles; 2–3 bristles also arising laterally from basal segment. Antenna nearly as long as forewing; scales smoothly appressed, dark brown dorsally, paler, more white ventrally; scape dark brown irrorated with white scales and with a row of white scales bordering distal margin; scales of scape and pedicel moderately broad; flagellum without cilia and with a single row of more slender scales completely encircling each segment.

*Thorax*: Forewing (Fig. 17) length 5–6 mm. Dorsum and tegula similar to head in color but with broader scales; thorax mostly pale cream ventrally. Forewing predominantly pale whitish cream, irrorated with scattered dark brown scales; 2–3 small patches of dark brown scales usually present on basal half of forewing along costal and dorsal margins and 2 patches within discal cell; fringe mostly light brown irrorated with dull white. Hindwing and fringe uniformly pale grayish brown; frenulum a single stout spine in male, not examined in female; M1 and 2 stalked ~ 0.6 their length. Fore and midlegs mostly light grayish brown dorsally and whitish cream ventrally with prominent dark brown banding on tibia and tarsomeres; hindleg generally paler in color.

*Abdomen*: Pale grayish brown dorsally, whitish cream ventrally. Eighth segment without coremata.

*Male genitalia* (Figs 24, 25): Segment 10 mostly sclerotized, fused to tegumen; uncus lobes minute, with caudal margin bifid. Tegumen a relatively narrow dorsal ring, with extended medium lobe fused to uncus. Vinculum broad, U-shaped, anterior margin broadly rounded, ~ 0.7× the length of valva. Valva simple; cucullus broad with rounded apex; costal margin densely setose. Juxta well developed as an elongate U-shaped pouch. Aedeagus slender, nearly as long as valva; vesica with numerous, minute spiculiform cornuti; base of aedeagus relatively deeply divided.

Female unknown.

#### Etymology.

The species name is derived from the genitive case of the type locality (Ascension).

#### Holotype.

♂, ASCENSION ISLAND: [Specific locality unknown] 4 Sept. 1958, E.A.G. Duffey, B.M. 1958-760, digital image captured, (BMNH).

#### Paratypes.

ASCENSION ISLAND: Same data as holotype: 6 ♂, BMNH genitalia slide ♂, 5854, BMNH wing slide 30835, (BMNH, NMNH).

#### Distribution

(Fig. 1). Ascension Island.

#### Biology.

Unknown; larvae are most likely plant detritvores or lichenivorous.

#### Remarks.

The species nearest to *Erechthias ascensionae*, both morphologically and geographically, is *Erechthias dracaenura* (Meyrick), which is known only from São Tomé, an island located off the western coast of central Africa in the Gulf of Guinea (Fig. 1). The forewing patterns of both species are similar (Figs 5, 6) in possessing a whitish background color irrorated with isolated brown scales and marked with 6–8 scattered, moderately large, darker brown to black spots. The forewings of *Erechthias dracaenura* generally appear more whitish and less heavily marked than those of *ascensionae*. The male genitalia of *Erechthias ascensionae* (Figs 24–25) differ from that of *Erechthias dracaenura* (Figs 26–27) in the apex of cucullus being more slender and in possessing a more elongate, tapered uncus.

### Erechthias grayi sp. n.

http://zoobank.org/06C8414F-FC41-4DD7-BAB9-CCA4F5B3BC83

http://species-id.net/wiki/Erechthias_grayi

Figs 7–10 , 18 , 28–31

#### Diagnosis.

**Adult** (Figs 7, 8). *Head*: Scales generally slender with bidentate apices; scales of frons smooth, appressed, directed dorsad, pale brown to gray on lower frons becoming dull white to pale gray at top of frons; scales of vertex erect or mostly so, especially over occipital tufts, fuscous, some with grayish white apices. Labial palpus with scales flattened and appressed dorsally, mostly dark grayish brown with scattered paler scales; venter of second segment with a dark brush of long, slender, erect scales and a lateral series of ~ 6-8 long, dark bristles; 1–2 bristles also arising laterally from basal segment. Maxillary palpus elongate, 5-segmented, approximately as long as labial palpus. Antenna ~ 1.6× the length of forewing; scales smoothly appressed, dark fuscous; scales of scape and pedicel moderately broad; flagellum without cilia and with a single row of more slender scales completely encircling each segment.

*Thorax*: Forewing brachypterous (Fig. 18), length 1.4–1.8 mm. Dorsum of thorax dark fuscous dorsally, with a few dull white scales at caudal margins of tegula and mesonotum; mostly grayish white ventrally. Forewing similar to dorsum in color, dark fuscous with an irregular scattering of dull white scales at base of wing and mostly crossing wing beyond middle; a slightly larger concentration of dull white scales at apex and extending a short distance along costa; fringe almost completely lacking, restricted to apex. Hindwing minute (Fig. 18), slightly variable in size, without scales; length ~ 0.15 mm; a single stout frenulum present in male ~ equal to length of hindwing (frenulum not examined in female); fringe absent. Fore and midlegs fuscous, lightly irrorated with pale grayish white scales; apices of tibia and tarsomeres ringed with grayish white; hindleg generally paler in color.

*Abdomen*: Dark fuscous dorsally, mostly grayish white ventrally. Eighth segment without coremata.

**Figures 28–31.**
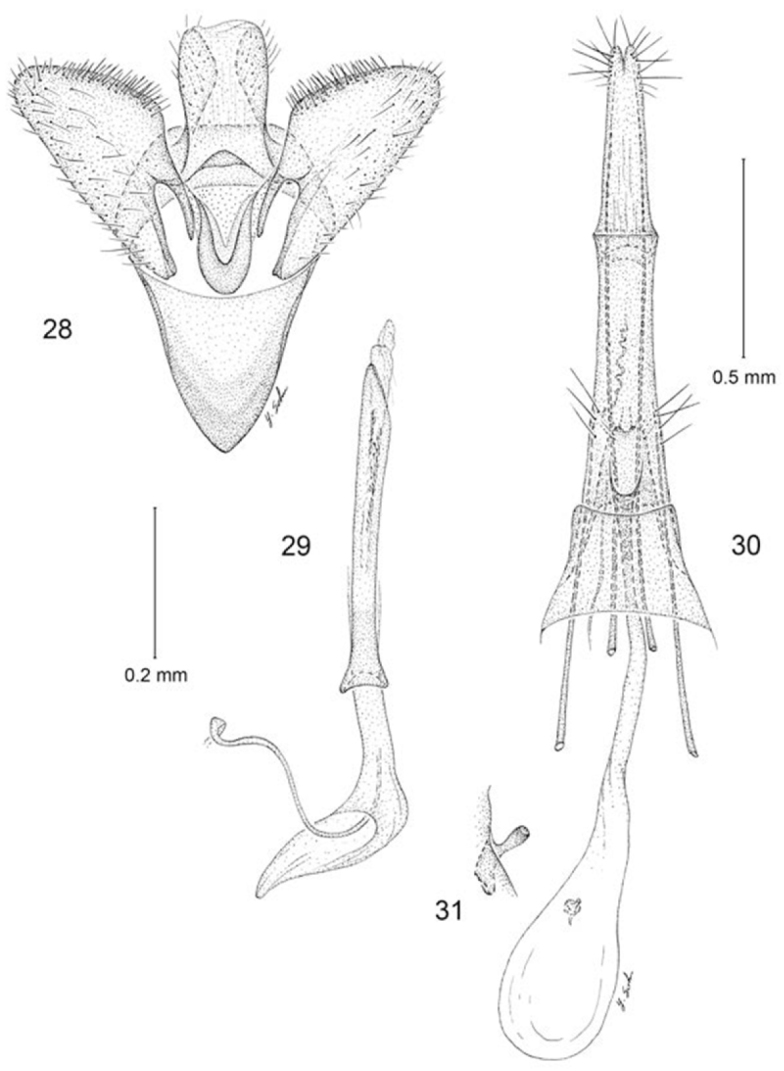
*Erechthias grayi*, genitalia **28** Male, ventral view(1 mm) **29** Aedeagus, ventral view **30** Female, ventral view **31** Detail of signum in Fig. **30**.

*Male genitalia* (Figs 28, 29): Segment 10 mostly membranous; uncus lobes indistinct, broadly rounded. Tegumen consisting of a relatively narrow dorsal ring. Vinculum broad, V-shaped, gradually tapering anteriorly with an acute anterior apex; vinculum ~ 0.7× the length of valva. Valva simple; cucullus broadly triangular with narrowly rounded apex; costal margin densely setose. Juxta well developed as an elongate U-shaped pouch. Aedeagus slender, ~ 1.3× length of valva; vesica with numerous, minute, spicular cornuti; base of aedeagus moderately flared, not divided.

*Female genitalia* (Figs 30, 31): Eighth sternite weakly sclerotized; ostium opening near anterior margin; an irregular cluster of ~ 5 pairs of long setae encircling caudal margin of eighth segment. Antrum slender, length ~ 3× maximum width. Ductus bursae slender, elongate, slightly longer than anterior apophysis, gradually enlarging to moderately large, ovate corpus bursae; walls of corpus bursae membranous except for very small, elongate signum; distal, more slender half of signum projecting beyond wall of corpus bursae.

#### Etymology.

The species name is a patronym for Alan Gray, a botanist who assisted Howard Mendel with the collection of this species on Ascension Island.

#### Holotype.

♂, ASCENSION ISLAND: Green Mountain, 743 m, Elliot’s Path, (Windy Corner), GPS 7.57S, 14.21W: 6 Aug. 2003, H. Mendel, BMNH(E) 2003-137, digital image captured (BMNH).

#### Paratypes.

ASCENSION ISLAND: same locality as holotype: 11 ♂, 1 ♀, 13 Dec. 2005, H. Mendel, BMNH slide 33642 ♀, BMNH(E) 2006-13; 4 ♂, 21 Nov. 2012, H. Mendel and A. Gray, USNM slide 34532 ♂. ASCENSION ISLAND: White Horse Hill [Little White Hill], S. E. Bay: 2 ♂, 23 Aug. 2012; [pitfall trap]; Ms L. White, USNM slide 34533 ♂. White Horse Rock [Little White Hill, S. E. Bay]: 2 ♂, 29 May 2013; running over lichen covered rock; pooter; leg. A. Wakeham-Dawson; digital image captured. (BMNH, USNM).

#### Distribution

(Fig. 1). Ascension Island. *Erechthias grayi* was at first thought to be confined to the higher elevations of Green Mountain where it was found on several occasions at altitudes around 743 m, in a very moist area frequently shrouded in cloud. Recent captures at Little White Hill, an extremely arid area at altitudes below 200 m show that *Erechthias grayi* is tolerant of a wide range of conditions. What the two areas do have in common is that the vegetation and habitat are comparatively undisturbed. Probably, *Erechthias grayi* would have been more widespread on Ascension prior to human habitation and the disturbances accompanying human settlement.

#### Biology.

Becauseadults have been collected in close association with lichen covered rocks, it is likely the larvae are lichenivorous, a frequently used food source in this group of moths. Several (13) small (3.5–4.2 mm long) mature larval cases, some with pupal exuviae attached (Figs 9, 10), were collected under lichen covered rocks at White Horse Hill, on the same day that an adult was collected there by A. Wakeham-Dawson. It is likely that these are the larval cases of *Erechthias grayi*, but larval rearings will need to be conducted to confirm this. The cases are mostly white, speckled with small grains of sand and minute, dark fragments from the rocky substrate.

The behavior of the moths in the field was unusual. They would cling firmly to the bare rock, lichens (Fig. 3), and small plants in exposed situations and were only seen to move when disturbed, and then reluctantly. They would hop a few inches in a very bug-like (Heteroptera) manner and were most easily collected using an aspirator (pooter). Wakeham-Dawson (see paratype data) observed adults running over a lichen covered rock at White Horse Hill.

#### Remarks.

*Erechthias grayi* is the only species within this large genus known to possess brachypterous adults, and thus is the most distinctive moth within *Erechthias*. The male and female genitalia of *Erechthias grayi* are most similar to those of *Erechthias darwini* Robinson (1983), one of the few species of Lepidoptera known to inhabit St. Paul’s Rocks (Pedro e Săo Paulo, Fig. 1), located slightly over 1000 miles northeast of Ascension Island. The male saccus of *Erechthias grayi* is more triangular and the female signum is more slender than those of *Erechthias darwini*. Most significantly, the adults of *Erechthias darwini* (Fig. 11) are fully winged and capable of flight. However, considering their genitalic similarities and geographical proximity, it is possible that *grayi* may have shared relatively recent common ancestrywith *darwini*.

Reportedly, some form of wing reduction has occurred in either 25 (Sattler 1991) or as many as 35 families (Heppner 1991) of Lepidoptera. The two family totals quoted probably differ because Sattler did not include species displaying only slight wing dimorphism, and also because of some differences in the family classification followed by each author. Because of rather obvious reasons involving different selective pressures and flight requirements that exist between the sexes, wing reduction has rarely evolved in male Lepidoptera. Most species that have developed wing reduction in both sexes occur on small oceanic islands or in restricted coastal habitats (Sattler 1991, Heppner 1991, 2010, Wagner and Liebherr 1992). Although several ecological and environmental factors can lead to a loss of flight among insects, the effect of continuous strong winds such as occur on isolated islands have long been considered a primary reason for wing reductions, particularly in Lepidoptera males (Sattler 1991, Wagner and Liebherr 1992). Previously, eight species representing five genera of Tineidae had been reported to exhibit some form of wing reduction, with four species (including three species of *Pringleophaga* Enderlein and one of *Proerodesma* Meyrick) being brachypterous in both sexes (Sattler 1991). No member of the large genus *Erechthias* had been reported as brachypterous prior to this report.

## Supplementary Material

XML Treatment for Erechthias

XML Treatment for Erechthias minuscula

XML Treatment for Erechthias ascensionae

XML Treatment for Erechthias grayi
